# The role of chemotherapy in advanced solitary fibrous tumors: a retrospective analysis

**DOI:** 10.1186/2045-3329-3-7

**Published:** 2013-05-11

**Authors:** Min S Park, Vinod Ravi, Anthony Conley, Shreyaskumar R Patel, Jonathan C Trent, Dina C Lev, Alexander J Lazar, Wei-Lien Wang, Robert S Benjamin, Dejka M Araujo

**Affiliations:** 1 Department of Sarcoma Medical Oncology Unit 0450, The University of Texas MD Anderson Cancer Center, 1515 Holcombe Blvd, Houston, TX 77030, USA; 2 Department of Cancer Biology and the Adult Sarcoma Research Center, The University of Texas MD Anderson Cancer Center, 1515 Holcombe Blvd, Houston, TX 77030, USA; 3 Department of Pathology and the Adult Sarcoma Research Center, The University of Texas MD Anderson Cancer Center, 1515 Holcombe Blvd, Houston, TX 77030, USA; 4 Current affiliation: Department of Hematology/Oncology, Minor and James Medical, PLLC, 515 Minor Avenue, Seattle, WA 98104, USA; 5 Current affiliation: Sarcoma Medical Oncology Program, University of Miami Sylvester Comprehensive Cancer Center, 1475 N.W. 12th Avenue, Miami, FL 33136, USA

**Keywords:** Solitary fibrous tumor, Chemotherapy, Doxorubicin, Hemangiopericytoma

## Abstract

**Background:**

Patients with advanced solitary fibrous tumors (SFTs) have a poor prognosis; treatment options for recurrent disease are particularly limited. Several novel targeted agents have recently shown promise against advanced SFTs, but the relative efficacy of new agents is difficult to assess because data on the efficacy of conventional chemotherapy for SFTs are limited. We thus sought to estimate the efficacy of conventional chemotherapy for SFTs by reviewing data on tumor response to therapy and progression-free survival from SFT patients who received this therapy.

**Methods:**

We retrospectively analyzed the clinical outcomes of 21 patients with grossly measurable, advanced SFTs (unresectable metastatic disease or potentially resectable primary tumors) who received conventional chemotherapy and follow-up at The University of Texas MD Anderson Cancer Center between January 1994 and June 2007. Best tumor response to therapy was assessed using the Response Evaluation Criteria In Solid Tumors 1.1. The Kaplan-Meier method was used to estimate median progression-free survival (PFS) duration.

**Results:**

Of 21 patients, 4 received more than 1 regimen of chemotherapy, for a total of 25 treatments. Doxorubicin-based chemotherapy was given in 15 cases (60%), gemcitabine-based therapy in 5 cases (20%), and paclitaxel in 5 cases (20%). First-line chemotherapy was delivered in 18 cases (72%). No patients had a complete or partial response, 16 (89%) had stable disease, and 2 (11%) had disease progression. Five patients (28%) maintained stable disease for at least 6 months after first-line treatment. The median PFS duration was 4.6 months. The median overall survival from diagnosis was 10.3 years.

**Conclusion:**

Conventional chemotherapy is effective in controlling or stabilizing locally advanced and metastatic SFTs. Our findings can serve as a reference for tumor response and clinical outcomes in the assessment of novel treatments for SFTs.

## Introduction

Solitary fibrous tumors (SFTs) are rare sarcomas considered to be of fibroblastic origin [1,2]. Hemangiopericytomas (HPC) are now considered to be a cellular variant of SFTs. SFTs commonly involve the lower extremities, lungs/pleura, abdominopelvic cavity and meninges but can be found anywhere [3-5]. The majority of SFTs behave in a relatively indolent fashion and are usually treated by surgical resection. The reported median 10-year overall survival (OS) rates for patients with SFTs range from 54% to 89% after complete surgical resection of localized disease [6-9]. However, for the approximately 20%–30% of patients with SFTs in whom local recurrences and/or distant metastases eventually develop, options for effective treatment are limited. SFTs are generally regarded as relatively chemoresistant tumors, but to date, the efficacy of systemic chemotherapy for SFTs has not been established in the literature [10].

Recently, several novel targeted agents have shown promising results in the treatment of advanced SFTs. Temozolomide-bevacizumab combination therapy [11] as well as sunitinib regimens [12] have produced tumor response and durable periods of disease stabilization in several patients with advanced SFTs. Sorafenib [13,14], pazopanib [15], and anti-insulin-like growth factor I agents [12,16] also have produced favorable results in some patients. However, it is difficult to assess the relative efficacy of these novel agents against SFTs without more definitive data on the efficacy of conventional cytotoxic chemotherapy regimens.

To estimate the efficacy of conventional cytotoxic chemotherapy for advanced SFTs, we evaluated tumor response and progression-free survival (PFS) duration in advanced SFT patients who received this therapy at a single institution during a 13-year period.

## Materials and methods

### Patient selection

This study was approved by the Institutional Review Board of The University of Texas MD Anderson Cancer Center, and informed consent was waived for the proposed patient record review.

We queried the soft-tissue tumor pathology database at MD Anderson Cancer Center to identify all patients whose tumor specimens had been evaluated by a sarcoma pathologist at our institution from January 1994 to June 2007 and who were diagnosed with SFTs. The clinical records of the patients identified by our database search were then reviewed using MD Anderson’s institutional electronic medical records database. These records included those of all clinical encounters at MD Anderson and all available outside records that had been scanned into the system. To be included in our retrospective analysis, patients had to have advanced SFTs with grossly measurable disease treated with systemic chemotherapy under the direction of an MD Anderson sarcoma medical oncologist, and patients’ available clinical records and radiologic scans were reviewed. Patients were considered to have advanced SFTs if they had unresectable metastatic disease or if they had primary tumors for which chemotherapy could improve local resectability. Excluded from our analysis were patients who had received adjuvant chemotherapy and who had no grossly measurable disease, patients who had been seen only at their initial visit without subsequent follow-up visits, and patients younger than 18 years of age at diagnosis.

### Clinicopathologic variables

We collected data on patient characteristics, including age at diagnosis, sex, ethnicity, and vital status as of April 1, 2010, and on primary tumor characteristics, including histologic diagnosis (SFT), site, size, and presence of metastases. Tumor site and size were determined from patients’ pathology reports, operative reports, and radiologic scans or reports.

We recorded the chemotherapy schedule and the regimen used for each patient. For patients with unresectable disease who had received chemotherapy only, we recorded the chemotherapy regimen, the number of cycles, the best tumor response reached, and the date of disease progression. For patients for whom radiologic scans were available, information on the best tumor response achieved and disease progression was ascertained from direct measurement of the image on the radiologic scan; otherwise, the information was collected from radiologic or clinical reports.

The medical records showed that all patients who received chemotherapy were evaluated with radiologic scans at baseline, during treatment, and after treatment. We assessed radiologic response to treatment on available images following the Response Evaluation Criteria In Solid Tumors (RECIST) v.1.1 [17]. For our analysis, we classified radiologic response as complete response, partial response, stable disease, or progressive disease according to RECIST.

### Statistical analysis

Patient characteristics were summarized using medians and ranges for continuous variables and frequencies and percentages for categorical variables. We used the Kaplan-Meier method and SPSS software to estimate median PFS duration and median OS duration. PFS duration was defined as the time interval from the beginning of chemotherapy until radiologic evidence of disease progression as defined by RECIST or death from any cause. OS duration was defined as the time interval from initial diagnosis or initiation of chemotherapy until death from any cause. Survival data were updated on April 1, 2010, and data for patients who were still alive were censored from survival analysis at that point.

## Results

### Patient and disease characteristics

Our initial database search identified 128 patients diagnosed with SFTs, treated, and followed at MD Anderson from January 1994 to June 2007. Of these patients, 21 met our study inclusion criteria (had advanced SFTs with grossly measurable disease and were treated with systemic chemotherapy) and were included in our analysis.

The clinical characteristics of the patients (12 men and 9 women) are summarized in Table 1. The majority of the patients (81%) were white, and the median age was 56 years (range, 19–74 years). The most common primary tumor site was the abdomen/pelvis region. Seventeen (81%) of the 21 patients had unresectable metastatic disease. Four patients had locally advanced disease that was potentially surgically resectable and received neoadjuvant chemotherapy.

**Table 1:** Patient and disease characteristics for 21 patients with solitary fibrous tumors treated with conventional chemotherapy

**Patient characteristics**		
Age at start of treatment (years)	**Median**	**Range**
	56	19–74
	**n**	**%**
Sex		
Men	12	57
Women	9	43
Ethnicity		
White	17	81
Hispanic	3	14
Asian	1	5
Primary tumor site		
Abdomen/pelvis	9	43
Lung/pleura	4	19
Central nervous system/meninges	3	14
Extremities	2	9
Head or neck	1	5
Gluteal region	1	5
Posterior trunk	1	5
Stage of disease		
Primary locally advanced	3	14
Locally recurrent	1	5
Metastatic	17	81
Prior regimens of chemotherapy		
0	18	86
1	3	14

### Chemotherapy regimens and tumor responses

The chemotherapy regimens used for patients in our retrospective analysis are listed in Table 2. Four patients received 2 different regimens of chemotherapy, and treatment history and tumor response were recorded separately for each regimen of chemotherapy, bringing the total number of chemotherapy treatments to 25. Chemotherapy was administered as a first-line treatment in the majority (18/25 [72%]) of cases. Six (24%) patients received second-line chemotherapy, and 1 patient received third-line chemotherapy.

**Table 2:** Conventional chemotherapy regimens used for study patients with solitary fibrous tumors (n = 25)*

**Chemotherapy regimen**	**n**	**%**
Doxorubicin based	15	60
Doxorubicin + ifosfamide	12	
Doxorubicin + dacarbazine	1	
Doxorubicin + cisplatin	1	
Doxorubicin	1	
Gemcitabine based	5	20
Gemcitabine + docetaxel	1	
Gemcitabine	4	
Paclitaxel	5	20

*Four patients were treated with 2 different regimens of chemotherapy, resulting in a total of 25 treatments.

Fifteen patients received doxorubicin-based therapy, with 14 patients receiving 75 mg/m^2^ of doxorubicin and 1 patient receiving 60 mg/m^2^ of doxorubicin. Doxorubicin was most often administered with 10 g/m^2^ of ifosfamide (in 12 of 15 cases). In all 15 patients, doxorubicin-based chemotherapy was the first-line treatment. Those patients not receiving doxorubicin-based therapy were prescribed gemcitabine-based chemotherapy (5 cases) and paclitaxel (5 cases).

The tumor responses to each type of regimen and to each line of chemotherapy are summarized in Table 3. No patient had a complete response or partial response to chemotherapy, regardless of the regimen used. Overall, 16 (89%) of 18 patients treated with first-line chemotherapy had stable disease, and 5 (31%) of these 16 patients had stable disease for longer than 6 months. Two patients (11%) exhibited disease progression during first-line chemotherapy.

**Table 3:** Response evaluation criteria in solid tumors responses to conventional chemotherapy in patients with advanced solitary fibrous tumors (n = 25)*

**Chemotherapy**	**Response to chemotherapy n (%)**	
	**Stable disease**	**Progressive disease**
First-line therapy (n = 18)	16 (89%)	2 (11%)
All doxorubicin-based therapies	14	1
Doxorubicin + ifosfamide	11	1
Doxorubicin + dacarbazine	1	0
Doxorubicin + cisplatin	1	0
Doxorubicin	1	0
All gemcitabine-based therapies	1	1
Gemcitabine + docetaxel	0	1
Gemcitabine	1	0
Paclitaxel	1	0
Second-line therapy (n = 6)	4 (67%)	2 (33%)
Gemcitabine	1	1
Paclitaxel	3	1
Third-line therapy (n = 1)	0 (0%)	1 (100%)
Gemcitabine	0	1
All lines of therapy (n = 25)	20 (80%)	5 (20%)

*Four patients were treated with 2 different regimens of chemotherapy, resulting in a total of 25 treatments.

Of the 6 patients who were treated with second-line chemotherapy, 4 (67%) had stable disease, with 1 maintaining stable disease for longer than 6 months, and 2 (33%) exhibited disease progression. The patient treated with third-line chemotherapy exhibited disease progression after 2 cycles of chemotherapy.

### PFS duration and OS duration

**Figure 1:**
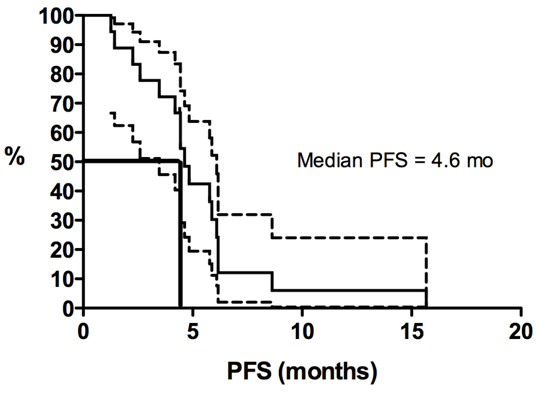
**Kaplan-Meier curves for progression-free survival duration (PFS) in patients with advanced solitary fibrous tumors who were treated with first-line conventional chemotherapy.** Y-axis represents the proportion of living patients without disease progression.

**Figure 2:**
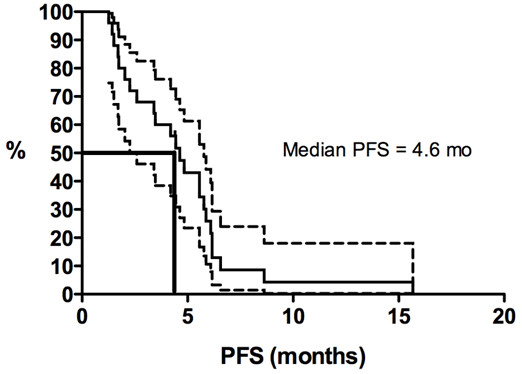
**Kaplan-Meier curves for progression-free survival duration (PFS) in patients with advanced solitary fibrous tumors who were treated with conventional chemotherapy (all lines of chemotherapy combined).** Y-axis represents the proportion of living patients without disease progression.

**Figure 3:**
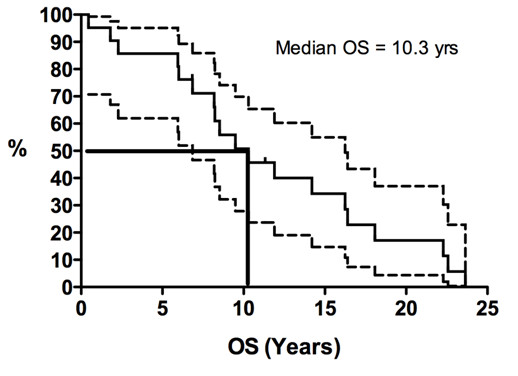
**Kaplan-Meier curves for overall survival duration (OS) from the time of diagnosis in patients with advanced solitary fibrous tumors who were treated with conventional chemotherapy.** Y-axis represents the proportion of living patients.

**Figure 4:**
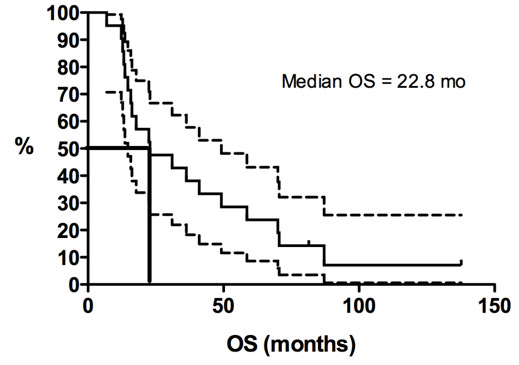
**Kaplan-Meier curves for overall survival duration (OS) from the initiation of chemotherapy in patients with advanced solitary fibrous tumor.** Y-axis represents the proportion of living patients.

Kaplan-Meier curves for PFS duration and OS duration are shown in Figures 1, 2, 3 and 4. The median PFS duration for the 18 patients who underwent first-line chemotherapy was 4.6 months (95% confidence interval [CI] = 4.0–5.3 months; Figure 1). The median PFS duration for the entire group was also 4.6 months (95% CI = 3.7–5.6 months; Figure 2). The median OS duration for all patients from diagnosis was 10.3 years (95% CI = 5.7–14.9 years; Figure 3), and the median OS duration from the start of chemotherapy was 22.8 months (95% CI = 3.1–42.6 months; Figure 4).

Most patients in the group died from progression of their disease. Two patients, however, were still alive at the time of this analysis on April 1, 2010. The first of these was a 28-year-old woman with an SFT in the right gluteal region who presented with a solitary metastasis in her right ilium. She underwent 4 cycles of neoadjuvant chemotherapy with intra-arterial doxorubicin (75 mg/m^2^) and cisplatin (120 mg/m^2^) for the metastatic tumor. Restaging studies after chemotherapy showed stable disease, and the patient underwent surgical resection of the tumor, which showed 50% necrosis. She received no further therapy and remained alive with no further evidence of recurrent disease after 11 years.

The second surviving patient was a 50-year-old man with a locally advanced SFT of the pleura. He received 4 cycles of neoadjuvant chemotherapy with doxorubicin (75 mg/m^2^) and ifosfamide (10 g/m^2^). He demonstrated stable disease after cycle 2 of chemotherapy, but the disease progressed after cycle 4 and the tumor was surgically resected. No significant necrosis was seen. The patient received no further therapy and remained alive with no evidence of recurrence after 6.3 years.

## Discussion

In our patients with locally advanced, recurrent, or metastatic SFTs treated with cytotoxic chemotherapy, the tumor response to chemotherapy was poor. The objective response rate was 0%, although 28% of patients had disease stabilization for longer than 6 months. The median PFS duration, measured from the start of chemotherapy, was 4.6 months.

Our findings are similar to those of Constantinidou et al., who found that only 1 (8%) of 13 patients with SFTs treated with first-line doxorubicin- or ifosfamide-based chemotherapy demonstrated a partial response and that the median PFS duration was 4 months [18]. In our study, only 2 patients survived and were disease-free at last follow-up. Both patients had received neoadjuvant chemotherapy; in 1 patient the disease progressed, and in the other, the tumor exhibited only 50% necrosis. It is thus more likely that surgery, not chemotherapy, had a primary role in the successful outcomes of these patients.

Stacchiotti et al. recently published the results of response to chemotherapy in 31 patients with solitary fibrous tumor [19]. In this study all patients received an anthracycline and 23/31 received an anthracycline plus ifosfamide [19]. Both our study and Stacchiotti’s study [19] used RECIST to measure response. In Stacchioti’s study [19] there were more patients with either a partial response 6 (20%) or progression 16 (53%) while in our study the majority of patients (16 (72%) had stable disease. Median progression-free survival in both studies are similar, 4 months in Stachhioti’s study [19] vs. 4.6 months in ours. Overall the findings in the two studies indicate that there is some clinical benefit of chemotherapy in solitary fibrous tumors.

The findings of our retrospective analysis were limited by our small sample size and thus by a lack of statistical power. The heterogeneity of our patients’ disease characteristics was another potential limitation. We also were only able to use RECIST, not Choi criteria, to assess tumor response to conventional chemotherapy [17,20,21]. Owing to the limitations of the medical records, especially the inconsistencies in how radiologic studies had been performed and archived, a Choi response assessment was not feasible for most of the patients. Among the 4 patients whose Choi responses were measurable (all of whom had been treated with first-line doxorubicin-based chemotherapy), only 1 demonstrated a Choi partial response (a size reduction of 13% and a computed tomography density reduction of 35%), and the other 3 showed Choi stable disease. Had we been able to assess the entire group’s tumor responses according to the Choi criteria, it is possible that those response rates would have been higher than the RECIST response rates. The reason for this is that in the Choi criteria the definition of a PR is a decrease in tumor size of 10% or more or a decrease in tumor density of 15% or more. This is in contrast to RECIST where the definition of a PR is a decrease of 30% or more in the sum of lesions. One of the limitations of using RECIST is that we might be overlooking a potentially beneficial treatment secondary to where the cut off for response is placed, 30% in RECIST vs. 10% in the Choi criteria. However, the relative short PFS seen in our group suggests that ultimately, conventional chemotherapy is not particularly clinically effective regardless of the best tumor response achieved. Our findings are also in contrast to previously reported cases that did show tumor responses to doxorubicin-based therapy, and this discrepancy likely reflects our study’s limited sample size [6,22].

Nevertheless, to our knowledge this retrospective analysis is one of the largest published series of patients with SFTs treated with conventional chemotherapy agents. The lack of objective tumor responses to chemotherapy observed in our patients suggests that treating patients who have advanced SFTs with first-line conventional doxorubicin- or gemcitabine-based chemotherapy might not be very effective to achieve tumor shrinkage. Thus, it may be useful to consider other therapies.

Recently, several novel agents have shown promise in the treatment of advanced SFTs. We previously reported our experience using the combination of temozolomide plus bevacizumab, a monoclonal antibody directed against the activity of vascular endothelial growth factor (VEGF) [11]. Fourteen patients with locally advanced, recurrent, or metastatic SFTs were treated, with 79% demonstrating a Choi partial response (i.e., tumor size reduction of ≥10%, computed tomography density reduction of ≥15%, or both) [20,21] and 14% demonstrating a RECIST partial response. The median PFS duration using the Choi response criteria was 9.7 months, and the median PFS duration using RECIST was 10.8 months.

Several tyrosine kinase inhibitors have also shown promise in treating SFTs. Sunitinib, a multitargeted tyrosine kinase inhibitor whose targets include VEGF receptors 1–3 and platelet-derived growth factor receptors A and B, has shown therapeutic promise for SFTs. Stacchiotti et al. found that 6 (60%) of 10 patients with advanced, chemotherapy-refractory SFTs treated with sunitinib achieved a Choi partial response, and 5 maintained their response for more than 6 months. The tumor response rate to sunitinib when assessed using RECIST was 0% [12]. Sorafenib, another anti-VEGF-receptor, multitargeted tyrosine kinase inhibitor, has also produced tumor responses in patients with SFTs [13,14]. Insulin-like growth factor I is overexpressed in some SFTs, and treatment regimens using figitumumab, an anti-insulin-like growth factor I receptor monoclonal antibody, have also produced tumor responses in a few patients with advanced SFTs [12,16]. These and other treatments might be better alternatives for the treatment of SFTs than conventional chemotherapy.

## Conclusions

Our findings indicate that conventional chemotherapy is effective in controlling or stabilizing locally advanced and metastatic SFTs. Our data on the clinical outcomes with traditional chemotherapy can serve as a reference point for comparing the efficacy of novel agents against SFTs. Despite the small number of patients treated with the temozolomide-bevacizumab combination and the sunitinib regimen to date, these agents appear to have greater efficacy than conventional chemotherapy for SFTs [11,12]. SFTs may be regulated by several different molecular pathways that could serve as potential targets for treatment, including (but certainly not limited to) those involving platelet-derived growth factor receptors, VEGF receptors, and insulin-like growth factor receptors [12-16]. One reasonable approach is to adopt targeted therapies/anti-angiogenesis drugs initially and then include chemotherapy later for disease stabilization. Additional molecular and clinical studies are needed to better understand this rare disease and to develop more effective treatments for it.

## Competing interests

The authors declare that they have no competing interests.

## Authors’ contributions

MP wrote the manuscript, VP performed the statistical analysis, AC helped write the manuscript, SP, RS, provided some of the patient cases, JC, DL, gave scientific advice about the project, AL, WLW confirmed the pathologic diagnosis and DA helped write and edit the manuscript. All authors read and approved the final manuscript.
